# The Vaccination Commission

**Published:** 1896-03-14

**Authors:** 


### The Vaccination Commission.

Some questions which deeply agitate the public mind are such in their own nature that they are not capable of being settled by the human reason. Of this class vaccination is not one. But though vaccination is admittedly a material and tangible question, the facts and results of which can be ascertained by the best methods of modern physical science, and which can, therefore, be definitely settled by mere weight of evidence, yet it is felt that all the best methods of modern science had not been competently applied to it when the reference to a Royal Commission was made, and it is perceived, therefore, that the Royal Commission which has been sitting for so many years on the subject has been amply justified in continuing its labours, notwithstanding the repeated calls made on it for a report. It would not have been justified if in response to the crude clamours of persons unaccustomed to deal with scientific evidence it had issued an inadequately considered report, the conclusions of which might be set aside by a single scientific discovery such as modern science has taught us to expect at her hands.

In reply to a question in the House of Commons last week, Sir Matthew White Ridley stated that "The Vaccination Commission last sat to take evidence orally on December 13th, 1893, but that since then they had procured and considered much valuable written evidence. . . . They have had twelve meetings for the consideration of their report, besides informal meetings of various members for the elucidation of particular points before their consideration by the general body of Commissioners. . . . They have given their labour ungrudgingly to the work of the Commission, and they are most earnestly desirous to bring their labours to a close." In a word, the Commissioners are thoroughly alive to the fact that they have undertaken a task which bristles with difficulties alike of a scientific, a political, and a practical character, and they will be thankful when their work is done, and the worry inseparable from the publication of their report is finally got rid of.

The medical profession, which necessarily knows more about the subject than any other portion of the community, will, we venture to predict, abide loyally by the conclusions and recommendations of the Commission. It can hardly be doubted that the report will confirm the general doctrine that efficient and repeated vaccination is a prophylactic against small-pox of the most valuable and demonstrable kind. If, pushing such a conclusion to its logical issue, the Commission should recommend more thoroughness in compulsion, and the more extensive and even compulsory adoption of re-vaccination, the medical profession, strong in its faith in prophylaxis, and in the value of sound health to the population, will no doubt accept such conclusions and give every assistance in carrying out the law, in spite of all the abuse and obloquy which a defeated and ignorant fanaticism may pour upon it. But it cannot be too perseveringly insisted upon that the efficiency of vaccination is one thing and the compulsory employment of the prophylactic is another and quite a different thing. The two have absolutely no connection with each other except such as has been, or may be, made by the Legislature. It is a mere question of policy, and accepting the lesser evil. Doctors, therefore, need no more stand for compulsion than any other class of men. If they do stand for compulsion, it is simply because, seeing in it a valuable aid to the nation's health, their patriotism cannot be content unless and until the nation makes use of that aid to the fullest possible extent.

If the Royal Commission, by sitting a little longer, can initiate a scheme which will take the sting out of compulsion, it will deserve well of the State, and will probably find its report as generally popular as its sittings have been judiciously prolonged.

